# The Control of the American Leafhopper *Erasmoneura vulnerata* (Fitch) in European Vineyards: Impact of Synthetic and Natural Insecticides

**DOI:** 10.3390/insects12020085

**Published:** 2021-01-20

**Authors:** Paola Tirello, Enrico Marchesini, Pamela Gherardo, Damiano Raniero, Filippo Rossetto, Alberto Pozzebon, Carlo Duso

**Affiliations:** 1 Department of Agronomy, Food, Natural resources, Animals and Environment, University of Padova, Viale dell’Università 16, Agripolis, Legnaro, 35020 Padova, Italy; paola.tirello@unipd.it (P.T.); pamela.gherardo@studenti.unipd.it (P.G.); damiano.raniero@studenti.unipd.it (D.R.); filippo.rossetto.1@studenti.unipd.it (F.R.); alberto.pozzebon@unipd.it (A.P.); 2 AGREA S.r.l. Centro Studi, Via Garibaldi 5/16, San Giovanni Lupatoto (VR), 37057 Verona, Italy; enrico.marchesini@agrea.it

**Keywords:** grapevine, *Vitis vinifera*, leafhoppers, vineyard management, chemical control

## Abstract

**Simple Summary:**

*Erasmoneura vulnerata*, a Nearctic leafhopper occurring on grapevine which is rarely damaging in North America, has become a new pest in European vineyards. Winegrowers are worried because of severe leaf symptoms potentially associated with yield losses and the nuisance posed when large numbers of adults occur at harvest time. Outbreaks were detected in conventional vineyards despite the use of broad-spectrum insecticides as well as in organic vineyards treated with pyrethrins. Therefore, the identification of effective control tools is required. Studies on *E. vulnerata* phenology have found that the second generation produces the largest population densities. We planned field trials to establish the most effective insecticides to be applied in conventional and organic vineyards. The most effective synthetic insecticides were acetamiprid, flupyradifurone and lambda-cyhalothrin, while the most effective natural product was kaolin.

**Abstract:**

The American leafhopper *Erasmoneura vulnerata*, detected in Europe in the early 2000s, has recently become a pest in North-Italian vineyards. Infestations were recorded in organic and conventional vineyards despite the application of insecticides targeting other pests. *Erasmoneura vulnerata* completes three generations per year, and the second generation is frequently associated with large populations. The selection of appropriate active ingredients and the timing of their application is crucial for effective pest control. Field trials were carried out in Northeastern Italy, using a randomized design, to evaluate the impact of insecticides applied against other grapevine leafhoppers on *E. vulnerata* populations. The beginning of the second generation was selected as the best time for insecticide application. For natural products, two applications were planned. Among the selected insecticides, the most effective were acetamiprid, flupyradifurone and lambda-cyhalothrin. Regarding natural products, the most effective was kaolin which could represent an alternative to pyrethrins in organic vineyards. The identification of pest threshold levels and the evaluation of side effects of the most effective insecticides on key natural enemies occurring in vineyards are required.

## 1. Introduction

The most important leafhoppers in European vineyards are *Empoasca vitis* (Göthe) and *Scaphoideus titanus* Ball. *Empoasca vitis* has been considered a pest in France, Italy, Switzerland, and other countries [1,2,3,4,5,6,7]. Traditionally, its control has been achieved using insecticides, sometimes specific (e.g., pyrethroids in France) or aimed at the control of berry moths and leafhoppers (e.g., organophosphates and chitin-inhibitors in Italy and Switzerland) [8,9,10]. In the 1990s, organophosphates’ effectiveness in controlling *E. vitis* declined, probably because of the selection of strains resistant to pesticides [11]. At the same time, research showed that *E. vitis* populations are limited by a number of natural enemies, namely the Hymenoptera Mymaridae (e.g., [12,13,14,15,16]). These findings, improved knowledge on cultivar susceptibility, and the adoption of action thresholds has reduced the attention towards *E. vitis*.

*Scaphoideus titanus* is the main vector of phytoplasma strains of the elm yellows group (16SrV) involved in the Flavescence dorée, a destructive disease of European vineyards [17,18,19]. In the 1990s, Flavescence dorée phytoplasma was declared a quarantine pest by the EU and control measures were made mandatory in France and Italy. Although chemical control is considered crucial in this framework, issues with Flavescence dorée spread [20,21,22]. In contrast to *E. vitis*, the efficacy of organophosphates towards *S. titanus* has remained satisfactory [11] and thus, resistance is not a concern for this pest. Problems with Flavescence dorée are serious in organic vineyards where growers can use only natural products (e.g., pyrethrins) characterized by limited activity and persistence [23,24]. Recently, new compounds replaced organophosphates in most viticultural areas and some of them (e.g., neonicotinoids) were very effective against leafhoppers [25,26,27]. However, in the last two years, several active ingredients have been banned in Europe and some of the remaining insecticides showed lower effectiveness against leafhoppers occurring in vineyards. The selection of active ingredients has become more important than in the past and chemical control must be integrated with agronomic and cultural measures to obtain adequate control of grapevine leafhoppers [20,22,28].

In this context, the American leafhopper *Erasmoneura vulnerata* (Fitch) (Hemiptera: Cicadellidae), first detected in Europe in 2004 [29], has become a pest in vineyards [30]. Although earlier records considered this species very harmful in North America, recent findings ranked it as a minor pest in leafhopper communities [31,32]. Initially *E. vulnerata* was localized in unsprayed vineyards in Northeastern Italy, then it spread to Northwestern Italy, Slovenia and Switzerland [33,34,35]. Currently, outbreaks involve both conventional and organic vineyards located in Northeastern Italy, particularly in the Veneto region [36]. Winegrowers are worried because of severe symptoms (leaf discoloration and leaf fall) potentially associated with yield losses and the nuisance to grape pickers when large numbers of adults are active at harvest time. Issues with *E. vulnerata* have been detected, although insecticides were applied against *S. titanus*. Investigations on *E. vulnerata* biology, ecology and behavior have been planned to implement effective control measures. This pest can complete three generations per year. Overwintered adults can damage shoots at sprouting, but the first nymphal generation is usually not harmful. The second generation is associated with the highest population densities, while the third is sometimes a problem [36]. We conducted field trials to evaluate the impact of insecticides used against other leafhoppers on *E. vulnerata* second generations. The results obtained in these trials are reported here.

## 2. Materials and Methods

The effects of a number of insecticides on *E. vulnerata* populations were evaluated in three conventional vineyards located in Vicenza and Verona provinces (Veneto region, Northeastern Italy) during the 2017, 2018 and 2019 growing seasons. In 2017, trials were carried out in a vineyard located in the Vicenza plain (Lonigo, cv. Garganega, Sylvoz training system, planting space 2.70 m × 1.40 m). In 2018, a hilly vineyard (Monteforte d’Alpone, cv. Trebbiano, Guyot training system, planting space 2.30 m × 0.9 m) located in the Verona province was selected for trials. The vineyard selected in 2019 (Colognola ai Colli, cv. Garganega, pergola veronese training system, planting space 3.70 m × 0.90 m) was also located in the Verona province. In these vineyards, the occurrence of *E. vulnerata* had been reported in the season preceding the study. Insecticides commonly applied in vineyards (active ingredients authorized in the EU and products authorized in Italy) and other products (e.g., kaolin) potentially useful for leafhoppers control were selected for trials (Table 1). An untreated control was included in each trial.

Trials were carried out according to a completely randomized design where each treatment comprised four replicates of 8–10 vines. Insecticides were applied (1 or 2 applications depending on label instructions) against the second generation of *E. vulnerata* (Table 1). Sampling was conducted before and after (3, 7, 10, 14 and 21 days) insecticide applications. A total of 40 leaves per treatment (10 leaves per replicate) were removed and transferred to the laboratory where leafhoppers were identified to species and stage (for *E. vulnerata*: I-II instar nymphs, III-V instar nymphs, adults) levels under a dissecting microscope.

Data were analyzed using a repeated measures linear mixed model with the MIXED procedure of SAS^®^ (ver. 9.3; SAS Institute Inc., Cary, NC, USA). Data obtained in each field trial were analyzed separately. In all models, the number of nymphs per leaf was considered as a response variable with repeated measures made at different times. Insecticide, time of sampling, and their interaction were considered as sources of variation in the model and tested using an *F* test (α = 0.05). Multiple comparisons of the abundance of *E. vulnerata* on different treatments were performed using *t*-test (α = 0.05) on the least-square means. The degrees of freedom were estimated with Kenward–Roger method, which can calculate non-integer values for error terms. Prior to the analysis, data were checked for model assumptions. The model was run on data transformed to log (n + 1), while untransformed data are shown in the figures. The SLICE option of the LSMEANS statement was used to test treatment effect variation during observation periods.

## 3. Results

### 3.1. 2017

**Figure 1:**
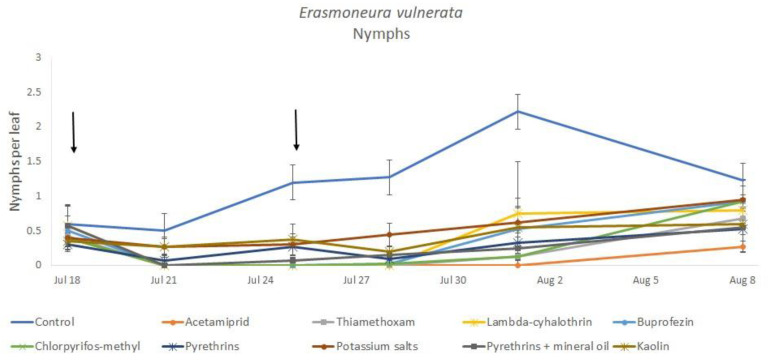
Dynamics of *Erasmoneura vulnerata* nymphs (mean ± std. err.) in the 2017 trial. Insecticides were applied one (first arrow) or two times (second arrow, see Materials and Methods section).

**Figure 2:**
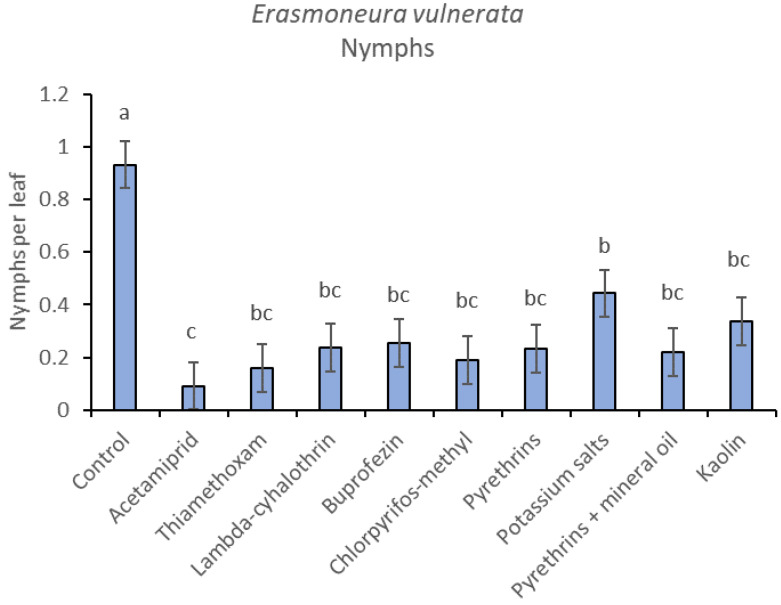
Least-square means (± std. err.) of *Erasmoneura vulnerata* nymphs observed on different treatments in the 2017 trial. Different letters indicate significant differences at *t*-test (α = 0.05).

Most of the leafhopper specimens found in leaf samples belonged to *E. vulnerata* nymphs. Adults of this species were rarely detected. Therefore, statistical analyses were carried out on total nymphs, early (I-II instar) nymphs and older (III-V instar) nymphs. In 2017, the effects of treatment and time were significant (for treatment: F = 2.78; d.f. = 9, 33.4; *p* = 0.015; for time: F = 14.29; d.f. = 5, 140; *p* < 0.0001). Interaction “treatment*time” was not significant (F = 1.07; d.f. = 45, 136; *p* = 0.367). No differences among treatments were found prior to the first insecticide application (F = 0.14; d.f. = 9, 97.6; *p* = 0.999). Among insecticides, acetamiprid was more effective than potassium salts (Figure 1 and Figure 2).

**Figure 3:**
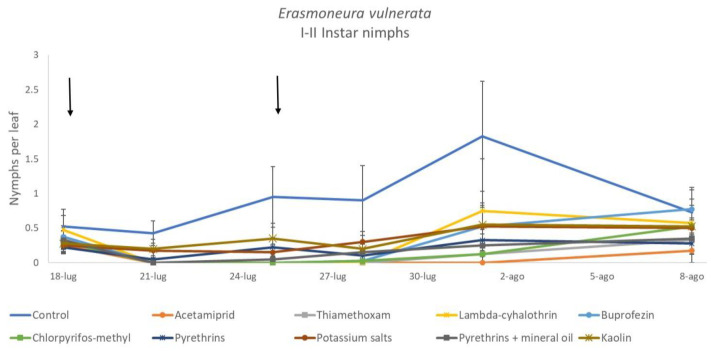
Dynamics of *Erasmoneura vulnerata* early (I-II instar) nymphs (mean ± std. err.) in the 2017 trial. Insecticides were applied one (first arrow) or two times (second arrow, see Materials and Methods section).

**Figure 4:**
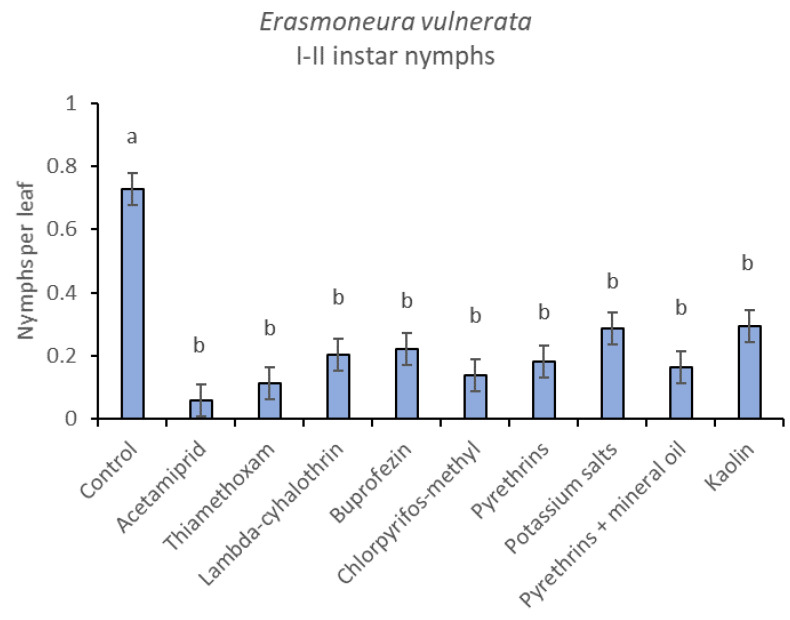
Least-square means (± std. err.) of *Erasmoneura vulnerata* early (I-II instar) nymphs observed on different treatments in the 2017 trial. Different letters indicate significant differences at *t*-test (α = 0.05).

**Figure 5:**
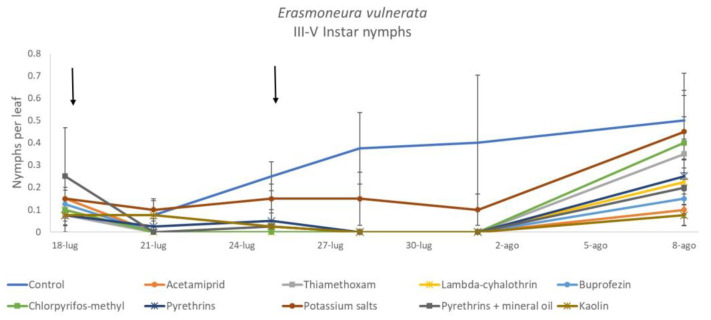
Dynamics of *Erasmoneura vulnerata* older (III-V instar) nymphs (mean ± std. err.) in the 2017 trial. Insecticides were applied one (first arrow) or two times (second arrow, see Materials and Methods section).

When early (I-II instar) and older (III-V instar) nymphs were considered separately, differences among treatments emerged only for the former (F = 3.03; d.f. = 9, 38.6; *p* = 0.008) (Figure 3, Figure 4 and Figure 5). In this comparison, there were no differences among insecticides (Figure 4). Considering older nymphs, there were no differences among treatments (F = 2.16; d.f. = 9, 27.1; *p* = 0.058). The effect of time emerged for early (F = 10.46; d.f. = 5, 143; *p* < 0.0001) as well as for older nymphs (F = 16.49; d.f. = 5, 127; *p* < 0.0001). Interaction “treatment*time” was not significant for early (F = 1.06; d.f. = 45, 140; *p* = 0.389) nor for older nymphs (F = 0.76; d.f. = 45, 126; *p* = 0.859).

### 3.2. 2018

**Figure 6:**
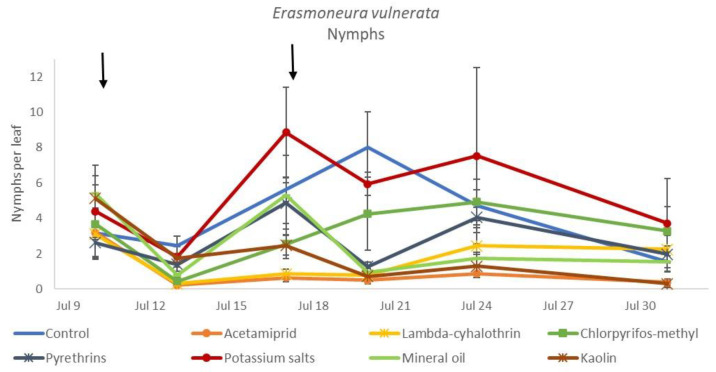
Dynamics of *Erasmoneura vulnerata* nymphs (mean ± std. err.) in the 2018 trial. Insecticides were applied one (first arrow) or two times (second arrow, see Materials and Methods section).

**Figure 7:**
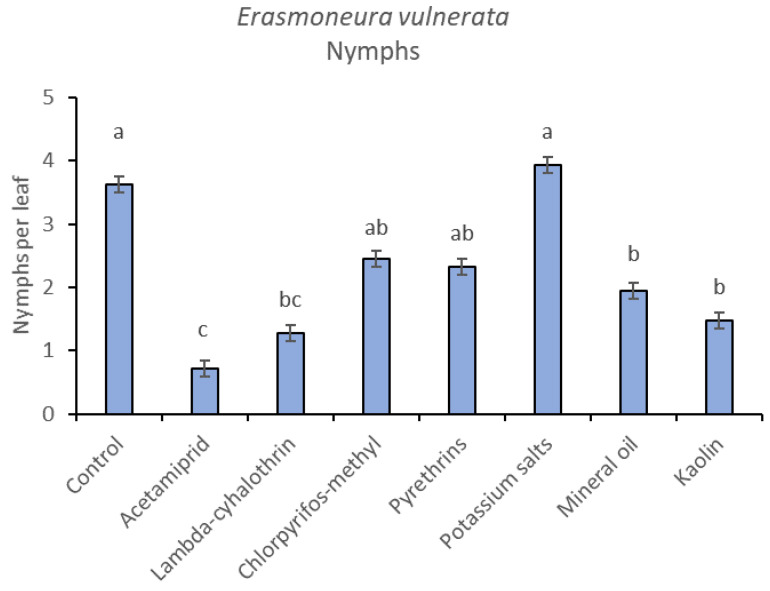
Least-square means (± std. err.) of *Erasmoneura vulnerata* nymphs observed on different treatments in the 2018 trial. Different letters indicate significant differences at *t*-test (α = 0.05).

In the 2018 trial, insecticide applications showed significant effects on *E. vulnerata* nymphs (F = 8.87; d.f. = 7, 26.2; *p* < 0.0001). Nymph densities fluctuated over sampling dates (F = 27.43; d.f. = 5, 101; *p* < 0.0001) and interaction “treatment*time” was significant (F = 2.75; d.f. = 35, 103; *p* < 0.0001) because the effect of treatment emerged after the first insecticide application onwards (Figure 6). There were no differences among treatments before insecticide application (F = 0.92; d.f. = 7, 106; *p* = 0.496). Higher *E. vulnerata* nymph densities were found in the control compared with the acetamiprid, lambda-cyhalothrin, mineral oil and kaolin treatments (Figure 7). Acetamiprid was more effective than mineral oil and kaolin. The application of chlorpyrifos-methyl, potassium salts and pyrethrins did not significantly decrease leafhopper densities compared with the untreated control.

**Figure 8:**
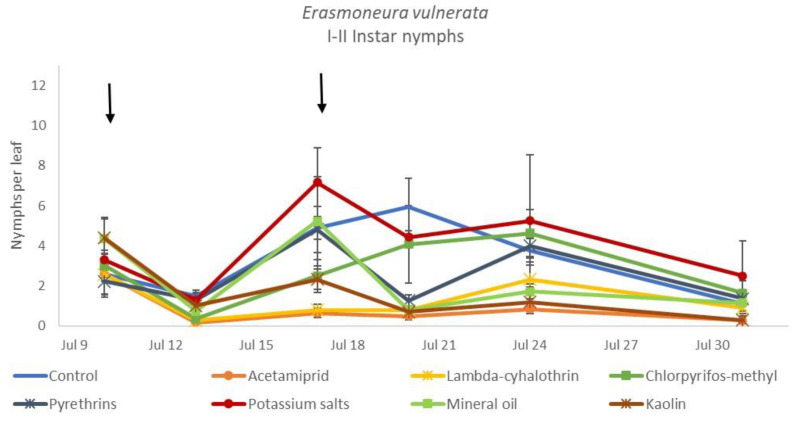
Dynamics of *Erasmoneura vulnerata* early (I-II instar) nymphs (mean ± std. err.) in the 2018 trial. Insecticides were applied one (first arrow) or two times (second arrow, see Materials and Methods section)**.**

**Figure 9:**
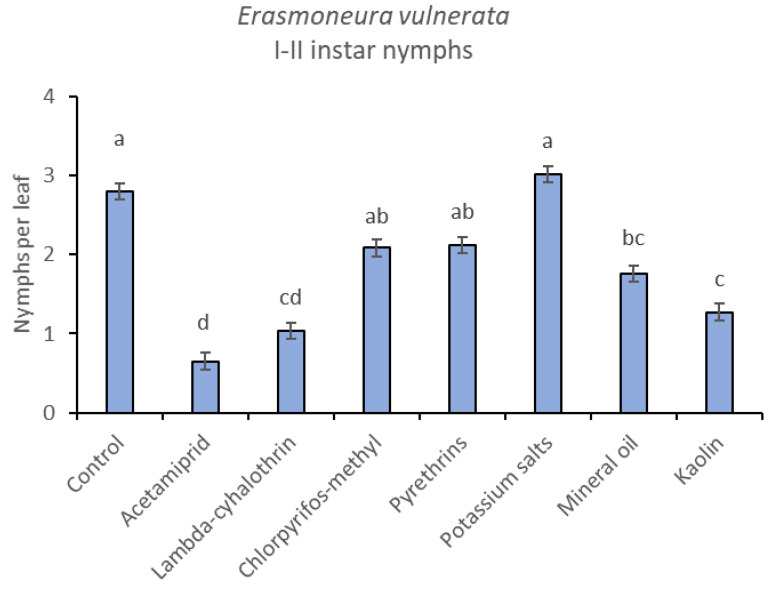
Least-square means (± std. err.) of *Erasmoneura vulnerata* early (I-II instar) nymphs observed on different treatments in the 2018 trial. Different letters indicate significant differences at *t*-test (α = 0.05).

**Figure 10:**
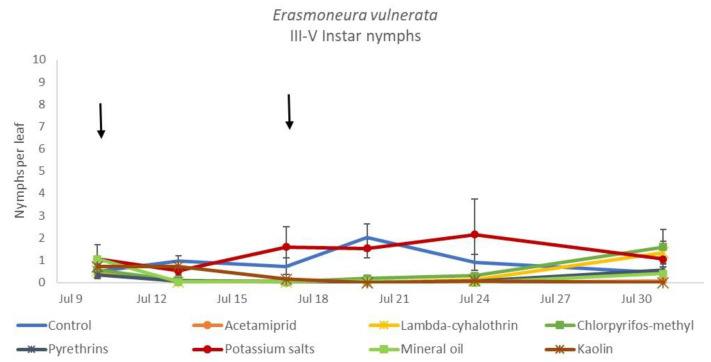
Dynamics of *Erasmoneura vulnerata* older (III-V instar) nymphs (mean ± std. err.) in the 2018 trial. Insecticides were applied one (first arrow) or two times (second arrow, see Materials and Methods section)**.**

**Figure 11:**
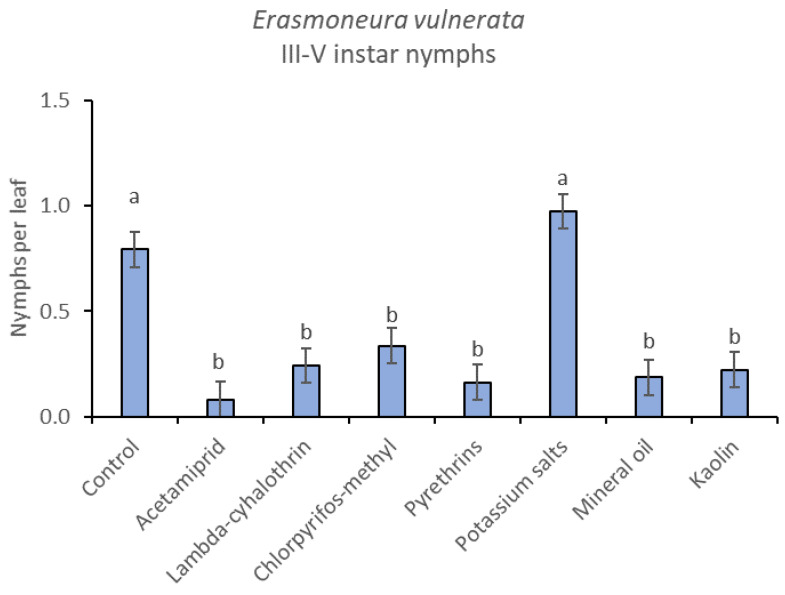
Least-square means (± std. err.) of *Erasmoneura vulnerata* older (III-V instar) nymphs observed on different treatments in the 2018 trial. Different letters indicate significant differences at *t*-test (α = 0.05).

The effects of treatment, time and their interaction were also significant when considering early (treatment: F = 9.61; d.f. = 7, 26.4; *p* < 0.0001; time: F = 35.72; d.f. = 5, 98.1; *p* < 0.0001; treatment*time: F = 2.6; d.f. = 35, 102; *p* = 0.0001) or older nymphs (treatment: F = 7.34; d.f. = 7, 26.3; *p* < 0.0001; time: F = 4.48; d.f. = 5, 101; *p* = 0.001; treatment*time: F = 2.26; d.f. = 35, 103; *p* = 0.0008) separately (Figure 8, Figure 9, Figure 10 and Figure 11). No differences among treatments were found prior to the first insecticide application when considering early (F = 1.07; d.f. = 7, 115; *p* = 0.384) and older nymphs (F = 0.54; d.f. = 7, 108; *p* = 0.803) separately, while differences among treatments emerged later. Considering early nymphs, acetamiprid and lambda-cyhalothrin were confirmed to be the most effective insecticides (Figure 9). In this comparison, kaolin was more effective than chlorpyrifos-methyl, pyrethrins and potassium salts (Figure 9). Considering older nymphs, all insecticides exception made for potassium salts reduced leafhopper numbers compared with the control (Figure 11).

### 3.3. 2019

**Figure 12:**
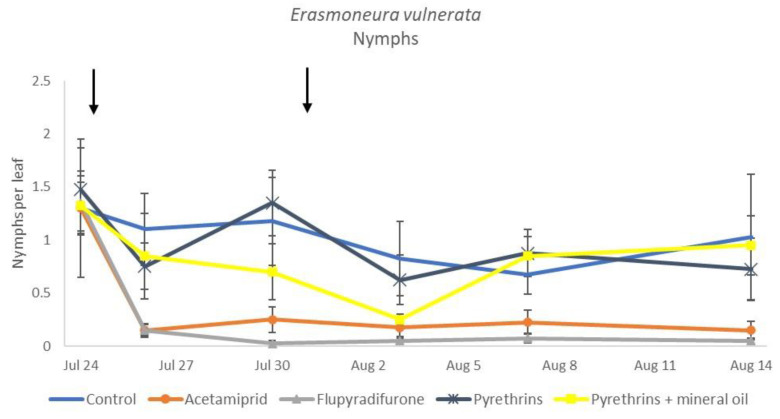
Dynamics of *Erasmoneura vulnerata* nymphs (mean ± std. err.) in the 2019 trial. Insecticides were applied one (first arrow) or two times (second arrow, see Materials and Methods section).

**Figure 13:**
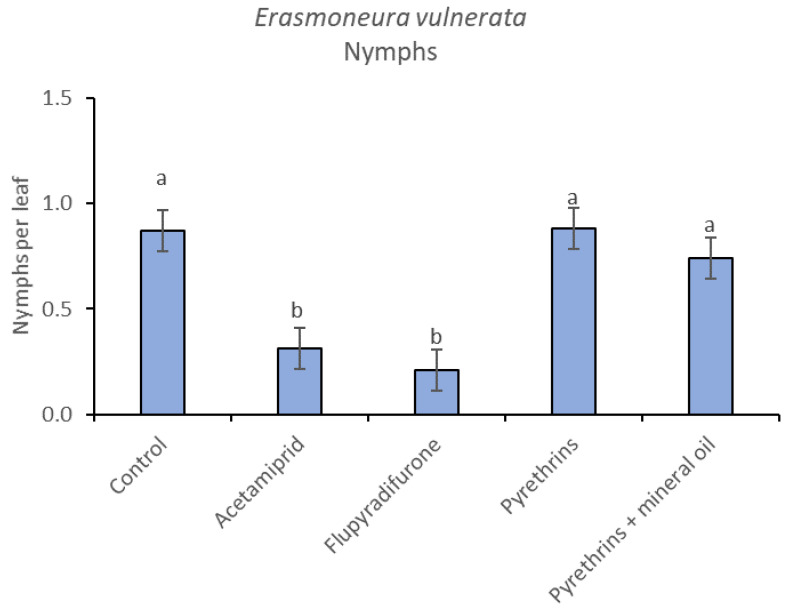
Least-square means (± std. err.) of *Erasmoneura vulnerata* nymphs observed on different treatments in the 2019 trial. Different letters indicate significant differences at *t*-test (α = 0.05).

In 2019, the effect of insecticides on *E. vulnerata* nymphs were confirmed to be significant (F = 5.17; d.f. = 4, 16.8; *p* = 0.007). Nymph densities changed over time (F = 13.7; d.f. = 5, 69.9; *p* < 0.0001) and a significant interaction “treatment*time” was found (F = 1.95; d.f. = 20, 69; *p* = 0.022). There were no differences among treatments before insecticide applications (F = 0.13; d.f. = 4, 47.8; *p* = 0.969), while differences emerged later (Figure 12). Acetamiprid and flupyradifurone significantly reduced *E. vulnerata* nymphs compared with the control, while pyrethrins and pyrethrins + mineral oil were not effective (Figure 13). The last two treatments did not differ significantly.

**Figure 14:**
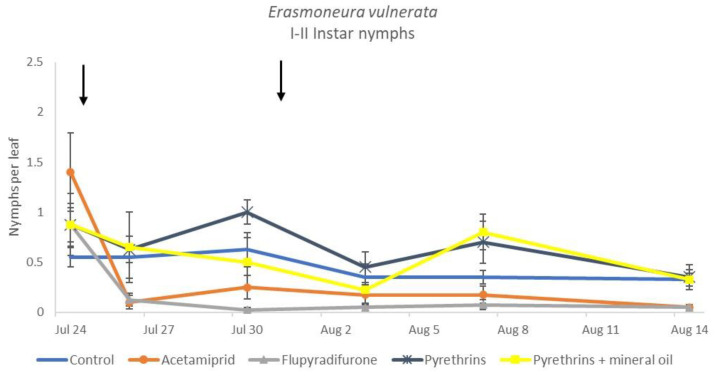
Dynamics of *Erasmoneura vulnerata* early (I-II instar) nymphs (mean ± std. err.) in the 2019 trial. Insecticides were applied one (first arrow) or two times (second arrow, see Materials and Methods section)**.**

**Figure 15:**
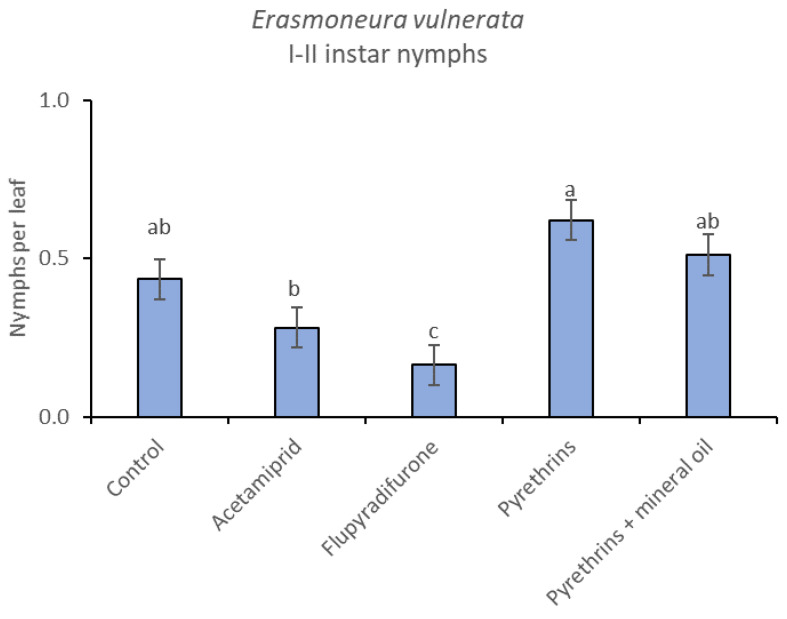
Least-square means (± std. err.) of *Erasmoneura vulnerata* early (I-II instar) nymphs observed on different treatments in the 2019 trial. Different letters indicate significant differences at *t*-test (α = 0.05).

**Figure 16:**
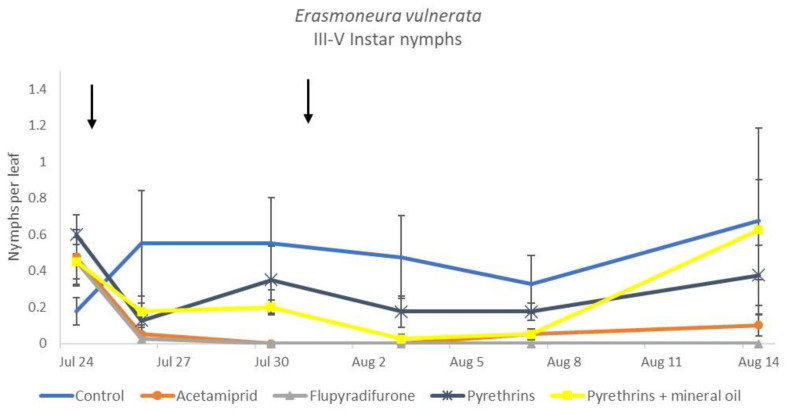
Dynamics of *Erasmoneura vulnerata* older (III-V instar) nymphs (mean ± std. err.) in the 2019 trial. Insecticides were applied one (first arrow) or two times (second arrow, see Materials and Methods section)**.**

**Figure 17:**
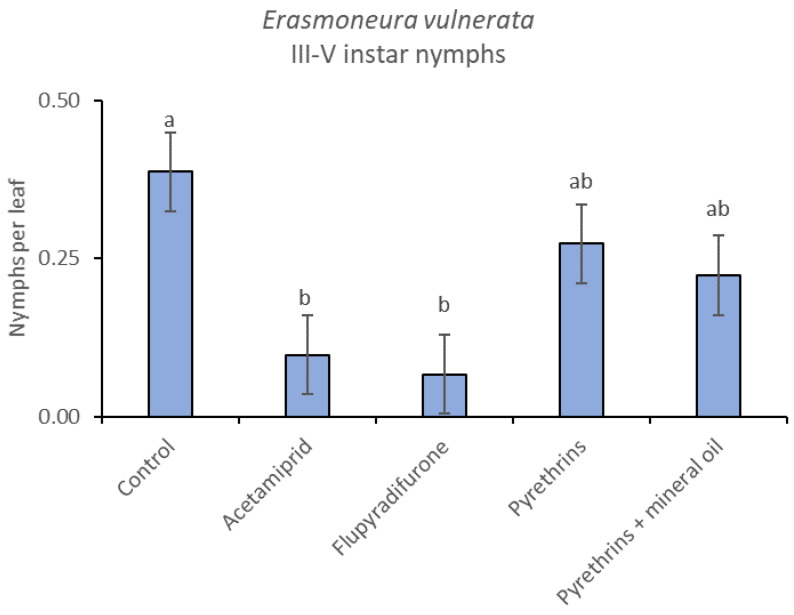
Least-square means (± std. err.) of *Erasmoneura vulnerata* older (III-V instar) nymphs observed on different treatments in the 2019 trial. Different letters indicate significant differences at *t*-test (α = 0.05).

When the statistical analysis was conducted on early or older nymphs, the effects of treatment, time and “treatment*time” were confirmed (for I-II instar nymphs-treatment effect: F = 4.59; d.f. = 4, 19.4; *p* = 0.009; time effect: F = 19.11; d.f. = 5, 71.6; *p* < 0.0001; treatment*time effect: F = 3.57; d.f. = 20, 70.7; *p* < 0.0001; for III-V instar nymphs-treatment effect: F = 3.18; d.f. = 4, 16.5; *p* = 0.041; time effect: F = 6.64; d.f. = 5, 67.8; *p* < 0.0001; treatment*time effect: F = 2.15; d.f. = 20, 67.4; *p* < 0.011) (Figure 14, Figure 15, Figure 16 and Figure 17). No differences among treatments were found prior to the first insecticide application (early nymphs: F = 2.21; d.f. = 4, 55.4; *p* = 0.08; older nymphs: F = 1.33; d.f. = 4, 53.6; *p* = 0.271), while the effect of treatment emerged later (Figure 14 and Figure 16). Flupyradifurone and acetamiprid significantly reduced the abundance of early and older nymphs (Figure 15 and Figure 17).

## 4. Discussion

Among the products tested in this study, those based on acetamiprid (IRAC Group 4A) were the most effective in controlling *E. vulnerata* populations in two out of three trials. In 2019, its impact was slightly lower than that of flupyradifurone (IRAC Group 4D), a novel insecticide belonging to the Butenolides and recommended against sucking pests. Its effectiveness against *E. vulnerata* is consistent with those reported in the control of *Erythroneura elegantula* Osborn and *E. ziczac* Walsh in North America [37]. A single application of these two insecticides, at the beginning of the second generation, maintained *E. vulnerata* population densities at low levels for some weeks. Another neonicotinoid (IRAC Group 4A), i.e., thiamethoxam, proved to be very effective against *E. vulnerata* in 2017. It was largely employed against sucking insects, e.g., *E. vitis* and *S. titanus* [25,26,27,38]. Thiamethoxam was banned from the EU because of the adverse impact on pollinators [39,40] and thus was excluded in 2018 and 2019 trials. Buprofezin (IRAC Group 16) was tested in 2017, showing good effectiveness, but it was also banned from the EU and thus excluded from further evaluations. Lambda-cyhalothrin (IRAC 3A Group) showed an effectiveness slightly lower than that of acetamiprid, but leafhopper populations seemed to recover faster in the respective plots. Results obtained using the organophosphate chlorpyrifos-methyl (IRAC Group 1B) are of particular interest as this insecticide has been widely used against grapevine leafhoppers in Italy [26,38,41]. It was effective against *E. vulnerata* in 2017 but not in 2018. Different vineyards were selected for these trials, and thus a variation in the susceptibility of leafhopper populations could explain the different results we obtained. It should be mentioned that the first outbreaks of *E. vulnerata* in Northern Italy were detected in vineyards frequently treated with chlorpyrifos-methyl. This observation suggests that resistance to insecticides could be a key factor explaining the unexpected outbreaks of this species [30]. It should be mentioned that the closely related chlorpyrifos-ethyl has been widely used in European vineyards against *S. titanus*, berry moths, and scales [20] and resistance in *E. vitis* was strongly suspected [11]. Chlorpyrifos-methyl and chlorpyrifos-ethyl have been banned from the UE in 2020 because of concerns for human health and thus study on leafhopper resistance to these insecticides was discontinued.

With regard to natural products, pyrethrin-based insecticides (IRAC Group 3A) are widely used against *S. titanus* and other leafhoppers in organic vineyards in France, Italy and Switzerland [20]. In the current study, the application of pyrethrins gave contrasting results in controlling *E. vulnerata*. In 2017, pyrethrins significantly reduced nymph densities compared with the control, in 2018 they showed some effectiveness on older nymphs only, while in 2019 they showed unsatisfactory results in controlling leafhoppers. Mineral oils were effective in 2018 and it was expected they could increase the impact of pyrethrins when mixed; this assumption was verified in 2017 but not in 2019. Potassium salts were slightly effective in 2017 but were associated with poor results in 2018. Finally, kaolin (an inert white clay not classified as an insecticide) significantly reduced *E. vulnerata* densities in 2017 and 2018. It was more effective than chlorpyrifos-methyl, pyrethrins and potassium salts against early nymphs in 2018 trial. Kaolin was active on some grapevine pests [42], particularly towards *E. vitis* and *Z. rhamni* [43]. Inhibition of feeding was the main mode of action through which kaolin affected leafhopper nymph populations. Timing in applying kaolin against *E. vulnerata* and mechanisms underlining its mode of action are worthy of study.

## 5. Conclusions

Recent outbreaks of *E. vulnerata* in Europe have caused concern for winegrowers and suggest the value of testing the impact of a number of conventional or natural insecticides on this species in vineyards. Among the insecticides tested, the most effective were acetamiprid, flupyradifurone and lambda-cyhalothrin. A single application of these compounds reduced leafhopper population densities to low levels for some weeks. Regarding natural products, the most effective was kaolin, which could represent an alternative to pyrethrins in organic vineyards and a complementary tool in conventional vineyards. The use of insecticides should be selected at the correct time and once threshold levels are exceeded. Our knowledge of the biology of *E. vulnerata* allowed us to identify the best timing for insecticide application (i.e., at the beginning of the second generation) but threshold levels have not been defined yet [44]. Finally, insecticides’ side effects on beneficials occurring in European vineyards should be determined to optimize integrated pest management (IPM) strategies [45,46]. Information on the side effects of many of these insecticides on predatory mites belonging to the Phytoseiidae family is available [47,48] but knowledge is limited for other important beneficials.

## Figures and Tables

**Table 1:** Insecticides selected for field trials.

Year	Active	Trade	Concentration	Dose	Group	Number of
	Ingredients	Mark				Applications
**2017**	Untreated control	-	-	-	-	-
	Acetamiprid	Epik SL	50 g/L	150 mL/hL	Neonicotinoids	1
	Thiamethoxam	Actara 25 WG	25%	20 g/hL	Neonicotinoids	1
	Lambda-cyhalothrin	Karate Zeon	9.40%	25 mL/hL	Pyrethroids	1
	Buprofezin	Applaud Plus	25.00%	200 g/hL	Thiadiazines	1
	Chlorpyrifos-methyl	Reldan LO	21.40%	150 mL/hL	Organophosphates	1
	Pyrethrins	Biopiren Plus	18.6 g/L	160 mL/hL	Pyrethrins	2
	Pyrethrins +	Biopiren Plus +	18.6 g/L	160 mL/hL	Pyrethrins +	2
	mineral oil	Chemol Plus	80%	500 mL/hL	Mineral oils	
	Potassium salts	Ciopper	455 g/L	150 mL/hL	Salts	2
	Mineral oil	Chemol Plus	80%	500 mL/hL	Mineral oils	2
	Kaolin	Surround	95%	4 Kg/hL	Kaolin	2
**2018**	Untreated control	-	-	-	-	-
	Acetamiprid	Epik SL	50 g/L	150 mL/hL	Neonicotinoids	1
	Lambda-cyhalothrin	Karate Zeon	9.40%	25 mL/hL	Pyrethroids	1
	Chlorpyrifos-methyl	Reldan LO	21.40%	150 mL/hL	Organophosphates	1
	Pyrethrins	Biopiren Plus	18.6 g/L	160 mL/hL	Pyrethrins	2
	Potassium salts	Ciopper	455 g/L	150 mL/hL	Organic salts	2
	Mineral oil	Chemol Plus	80%	500 mL/hL	Mineral oils	2
	Kaolin	Surround	95%	4 Kg/hL	Kaolin	2
**2019**	Untreated	-	-	-	-	-
	Acetamiprid	Epik SL	50 g/L	150 mL/hL	Neonicotinoids	1
	Flupyradifurone	Sivanto Prime	200 g/L	60 mL/hL	Butenolides	1
	Pyrethrins	Biopiren Plus	18.6 g/L	160 mL/hL	Pyrethrins	2
	Pyrethrins +	Biopiren Plus +	18.6 g/L	160 mL/hL	Pyrethrins +	2
	mineral oil	Oliocin	80%	500 mL/hL	Mineral oils	

## Data Availability

The data presented in this study are available from the corresponding author, upon reasonable request.

## References

[1] Vidano C. (1958). Le cicaline italiane della vite. Hemiptera Typhlocibinae. Boll. Zool. Agr. Bachic..

[2] Vidano C. (1963). Alterazioni provocate da insetti in *Vitis* osservate, sperimentate e comparate. Ann. Fac. Sci. Agric. Univ. Torino.

[3] Schvester D., Moutous G., Bonfils J., Carle P. (1962). Ètude biologique des cicadelles de la vigne dans le sud-ouest de la France. Ann. Epiphyt..

[4] Moutous G., Fos A. (1973). Influence des niveaux de populations de cicadelles de la vigne (*Empoasca flavescens* Fab.) sur le symptôme de la grillure des feuilles. Ann. Zool. Ecol. Anim..

[5] Cerutti F., Baumgärtner J., Delucchi V. (1988). Ricerche sull’ecosistema ‘vigneto’ in Ticino: I. Campionamento delle popolazioni di *Empoasca vitis* Goethe (Hom., Cicadellidae, Typhlocybinae). Mitt. Schweiz. Entomol. Ges..

[6] Pavan F., Pavanetto E., Duso C., Girolami V., Vidano C., Arzone A. (1988). Population dynamics of Empoasca vitis (Goethe) and Zygina rhamni (Ferr.) on vines in northern Italy. Proceedings of the 6th Auchenorrhyncha Meeting, Turin, Italy, 7–11 September 1987.

[7] Decante D., van Helden M. (2006). Population ecology of *Empoasca vitis* (Goethe) and *Scaphoideus titanus* (Ball) in Bordeaux vineyards: Influence of migration and landscape. Crop Prot..

[8] Pavan F., Picotti P., Girolami V. (1992). Strategie per il controllo di *Empoasca vitis* Goethe su vite. L’Informatore Agrar..

[9] Baillod M., Jermini M., Antonin P., Linder C., Mittaz C., Carrera E., Udry V., Schmid A. (1993). Stratégies de lutte contre la cicadelle verte de la vigne, *Empoasca vitis* (Goethe). Efficacité des insecticides et problématique liée à la nuisibilité. Rev. Suisse Vitic. Arboric. Hortic..

[10] Bonafos R., Serrano E., Auger P., Kreiter S. (2007). Resistance to deltamethrin, lambda-cyhalothrin and chlorpyriphos-ethyl in some populations of *Typhlodromus pyri* Scheuten and *Amblyseius andersoni* (Chant) (Acari:Phytoseiidae) from vineyards in the south-west of France. Crop Prot..

[11] Girolami V., Mori N., Marchesini E., Duso C. (2001). Organophosphate resistance in grape leafhoppers and IPM strategies. Redia.

[12] Vidano C., Arzone A., Arnò C. Researches on natural enemies of viticolous Auchenorrhyncha. Integrated pest control in viticolture. In Proceedings of the Meet of EC Experts’ Group.

[13] Cerutti F., Delucchi V., Baumgärtner J., Rubli D. (1989). Ricerche sull’ecosistema ‘vigneto’ in Ticino: II. La colonizzazione dei vigneti da parte della cicalina *Empoasca vitis* Goethe (Hom., Cicadellide, Typhlocybinae) e del suo parassitoide *Anagrus atomus* Haliday (Hym. Mymaridae), e importanza della flora circostante. Mitt. Schweiz. Entomol. Ges..

[14] Van Helden M., Decante D. (2001). The possibilities for conservation biological control as a management strategy against *Empoasca vitis*. Integrated control in viticulture. IOBC/WPRS Bull..

[15] Ponti R., Ricci C., Torricelli R. (2003). The ecological role of hedges on population dynamics of *Anagrus* spp. (Hymenoptera: Mymaridae) in vineyards of Central Italy. IOBC/WPRS Bull..

[16] Zanolli P., Martini M., Mazzon L., Pavan F. (2016). Morphological and Molecular Identification of *Anagrus* ‘*atomus*’ Group (Hymenoptera: Mymaridae) Individuals from Different Geographic Areas and Plant Hosts in Europe. J. Insect Sci..

[17] Schvester D., Carle P., Moutous G. (1961). Transmission de la flavescence dorée de la vigne par *Scaphoideus littoralis* Ball, (Homopt. Jassidae). Ann. Epiphyt..

[18] Angelini E., Negrisolo E., Clair D., Borgo M., Boudon-Padieu E. (2003). Phylogenetic relationships among Flavescence dorée strains and related phytoplasmas determined by heteroduplex mobility assay and sequence of ribosomal and nonribosomal DNA. Plant Pathol..

[19] Malembic-Maher S., Desqué D., Khalil D., Salar P., Bergey B., Danet J.-L., Duret S., Dubrana-Ourabah M.-P., Beven L., Ember I. (2020). When a Palearctic bacterium meets a Nearctic insect vector: Genetic and ecological insights into the emergence of the grapevine Flavescence dorée epidemics in Europe. PLoS Pathog..

[20] Chuche J., Thiery D. (2014). Biology and ecology of the flavescence dorée vector *Scaphoideus titanus*: A review. Agron. Sust. Develop..

[21] Alma A., Lessio F., Gonella E., Picciau L., Mandrioli M., Tota F. (2018). New insights in phytoplasma-vector interaction: Acquisition and inoculation of flavescence dorée phytoplasma by *Scaphoideus titanus* adults in a short window of time. Ann. App. Biol..

[22] Mori N., Pavan F., Lessio F., Alma A. (2020). Vettori dei giallumi della vite, fra vecchie e nuove conoscenze. L’Informatore Agrar..

[23] Gusberti M., Jermini M., Wyss E., Linder C. (2008). Efficacité d’insecticides contre *Scaphoideus titanus* en vignobles biologiques et effets secondaires. Rev. Suisse Vitic. Arboric. Hortic..

[24] Tacoli F., Mori N., Pozzebon A., Cargnus E., Da Vià S., Zandigiacomo P., Duso C., Pavan F. (2017). Control of *Scaphoideus titanus* with natural products in organic vineyards. Insects.

[25] Delaiti M., Angeli G., Sandri O., Tomasi C., Ioriatti C. (2005). Nuovi insetticidi per il contenimento della cicalina verde della vite. L’Informatore Agrar..

[26] Posenato G., Marchesini E., Mori N. (2006). Efficacia di thiamethoxam su *Empoasca vitis* a confronto con lambda-cyhalothrin, abamectina, indoxacarb e chlorpyrifos. Atti Delle Giornate Fitopatol..

[27] Pozzebon A., Pederiva M., Moret R., Duso C. (2011). The impact of a number of insecticides on *Empoasca vitis* populations in north-eastern Italy. IOBC/WPRS Bull..

[28] Pavan F., Mori N., Bressan S., Mutton P. (2012). Control strategies for grapevine phytoplasma diseases: Factors influencing the profitability of replacing symptomatic plants. Phytopathol. Mediterr..

[29] Duso C., Bressan A., Mazzon L., Girolami V. (2005). First record of the grape leafhopper *Erythroneura vulnerata* Fitch (Hom., Cicadellidae) in Europe. J. Appl. Entomol..

[30] Duso C., Borgo M., Pozzebon A., Mazzon L., Mori N., Pavan F., Fornasiero D., Marchesini E., Martinez-Sañudo I., Zanettin G. (2017). Vite: *Erasmoneura vulnerata*, una minaccia da valutare. L’Informatore Agrar..

[31] Olivier C., Vincent C., Saguez J., Galka B., Weintraub P.G., Maixner M. (2012). Leafhoppers and planthoppers: Their bionomics, pathogen transmission and management in vineyards. Arthropod Management in Vineyards.

[32] Zimmerman R., Kondratieff B., Nelson E., Sclar C. (1996). The life history of two species of grape leafhoppers on wine grapes in western Colorado. J. Kans. Entomol. Soc..

[33] Duso C., Moret R., Manera A., Berto D., Fornasiero D., Marchegiani G., Pozzebon A. (2019). Investigations on the grape leafhopper *Erasmoneura vulnerata* in North-eastern Italy. Insects.

[34] Seljak G. (2011). First record of the Nearctic leafhopper *Erasmoneura vulnerata* (Fitch, 1851) (Hemiptera, Cicadomorpha: Cicadellidae) in Slovenia. Acta Entomol. Slov..

[35] Rizzoli A., Battelli R., Conedera M., Jermini M. (2020). First record of *Erasmoneura vulnerata* Fitch, 1851 (Hemiptera, Cicadellidae, Typhlocybinae) in Switzerland. Alp. Entomol..

[36] Duso C., Zanettin G., Gherardo P., Pasqualotto G., Raniero D., Rossetto F., Tirello P., Pozzebon A. (2020). Colonization patterns, phenology and seasonal abundance of the Nearctic leafhopper *Erasmoneura vulnerata* (Fitch), a new pest in European vineyards. Insects.

[37] O’Hearn J.S., Walsh D.B. (2020). Evaluating the Toxicity of Candidate Organic and Conventional Insecticides on Western Grape Leafhopper and Virginia Creeper Leafhopper (Hemiptera: Cicadellidae) under Vineyard and Laboratory Conditions. J. Entomol. Sci..

[38] Lavezzaro S., Morando A., Gallesio G. (2006). Un quadriennio di prove di lotta contro la cicalina verde della vite in Piemonte. Atti Delle Giornate Fitopatol..

[39] Medina-Pastor P., Triacchini G. (2020). The 2018 European Union report on pesticide residues in food. EFSA J..

[40] Jactel H., Verheggen F., Thiéry D., Escobar-Gutiérrez A.J., Gachet E., Desneux N. (2019). Alternatives to neonicotinoids. Environ. Int..

[41] Mori N., Posenato G., Sancassani G., Tosi L., Girolami V. (1999). Insetticidi per il controllo delle cicaline nei vigneti. L’Informatore Agrar..

[42] Puterka G.J., Reinke M., Luvisi D., Ciomperik M.A., Bartels D., Wendel L., Glenn D.M. (2003). Particle film, Surround WP, effects on glassy-winged sharpshooter behavior and its utility as a barrier to sharpshooter infestations in grape. Plant Health Prog..

[43] Tacoli F., Pavan F., Cargnus E., Tilatti E., Pozzebon A., Zandigiacomo P. (2017). Efficacy and mode of action of kaolin in the control of *Empoasca vitis* and *Zygina rhamni* (Hemiptera: Cicadellidae) in vineyards. J. Econ. Entomol..

[44] Rossetto F. (2019). Effetti di insetticidi su *Erasmoneura vulnerata* e valutazioni sui danni causati dall’insetto. Second-cycle degree. Agricultural Science and Technology.

[45] Duso C., Van Leeuwen T., Pozzebon A. (2020). Improving the compatibility of pesticides and predatory mites: Recent findings on physiological and ecological selectivity. Curr. Opin. Insect Sci..

[46] Pozzebon A., Tirello P., Moret R., Pederiva M., Duso C. (2015). A fundamental step in IPM on grapevine: Evaluating the side effects of pesticides on predatory mites. Insects.

[47] Duso C., Ahmad S., Tirello P., Pozzebon A., Klaric V., Baldessari M., Malagnini V., Angeli G. (2014). The impact of insecticides applied in apple orchards on the predatory mite *Kampimodromus aberrans* (Acari Phytoseiidae). Exp. Appl. Acarol..

[48] Tacoli F., Cargnus E., Duso C., Pozzebon A., Tirello P., Pavan F. (2019). Side effects of kaolin and bunch-zone leaf removal on predatory mite populations (Acari: Phytoseiidae) occurring in vineyards. J. Econ. Entomol..

